# Voices in Motion: Using I-Poems to Uncover Undergraduate University Students’ Psychosocial Journey and Physical Activity Behaviours

**DOI:** 10.3390/ijerph22060901

**Published:** 2025-06-05

**Authors:** Chanté Johannes, James Reid, Nicolette Roman

**Affiliations:** 1 Centre for Interdisciplinary Studies of Children, Families, and Society, University of the Western Cape, Cape Town 7535, South Africa; 2 School of Education, University of Huddersfield, Huddersfield HD1 3DH, UK

**Keywords:** physical activity, psychosocial factors, I-poems, university, students, South Africa

## Abstract

Physical inactivity is a pervasive global public health concern, yet there is limited qualitative research exploring the psychosocial dimensions of physical activity (PA) among undergraduate students at a South African university. Therefore, this study aimed to explore students’ PA participation, by providing insights into the psychosocial factors that shape their experiences. Interviews were conducted with 18 undergraduate university students between July and August 2023. I-poems, a creative qualitative method, were created from interview transcripts by isolating sentences featuring the pronoun “I” and arranging them into poetic stanzas without altering their sequence. This approach amplifies the participants’ voices, offering an authentic window into their lived experiences. Data was coded using the Atlas Ti v.8 software and thematically analysed to generate common themes. The I-poems revealed rich, layered insights from students regarding the psychosocial aspects of PA, highlighting themes such as mental health, motivation and social support. By centring the participants’ “I” narratives, the method foregrounded their voices, enabling a deeper exploration of their embodied PA experiences. This study highlights the potential of I-poems as a creative qualitative method to explore the intricacies of students’ PA journeys. The findings highlight the importance of considering psychosocial factors in understanding PA engagement, offering valuable subjective perspectives for designing contextually relevant and university student-tailored interventions that are suitable.

## 1. Introduction

### 1.1. Physical Activity and Psychosocial Factors Among Undergraduate Students

Physical activity (PA) during university years plays a pivotal role in establishing habits for lifelong PA in adulthood [1]. However, PA participation continues to decline across universities globally. For example, students attending a university in South Africa (SA) were found to have high rates of being overweight (20%) and obese (9.5%) [2]. Similarly, another South African study reported that 110 female undergraduate students were categorised as sedentary, with high rates of overweight (37.3%) and obesity levels (17.3%) [3]. Similarly, a Canadian study reported that students are not meeting the World Health Organisation (WHO) PA Recommendations [4,5], which suggests that young adults, such as undergraduate university students, should participate in at least 150 min of moderate or 75 min of vigorous PA per week [6]. Nevertheless, participation in PA is a complex health behavioural process consisting of many psychosocial factors [7,8]. Psychosocial factors such as mental health, motivation and social support have previously been researched as having a significant relationship with PA engagement among undergraduate university students [9,10]. One study reported that PA was positively related to stress (r = 0.11, *p* < 0.05) and anxiety (r = 0.10, *p* < 0.05), motivational factors were positively related to psychological condition and others’ expectations [r = 0.10, *p* < 0.05] as well as depression and others’ expectations (r = 0.11, *p* < 0.05) [10]. Similarly, a recent study indicated that mental health concerns, such as depression (23.2%) and anxiety (40.6%), were extremely severe and limited PA behaviour, whereas social support, social media and recognition from others were facilitators [10]. This suggests that the psychosocial journey of an undergraduate university student influences their health behaviours and should be explored in future research [11]. Psychosocial factors among undergraduate students, especially within South African tertiary institutions, are multifaceted due to the diverse student population [12]. Therefore, many South African universities are not prepared to cater to the diverse needs of their students [13]. Scant research has been conducted focusing on students’ psychosocial vulnerable factors and how these vulnerable factors impact other aspects of life, such as their overall well-being [14]. Despite the recognised importance of these psychosocial factors for PA engagement, and ultimately the enhancement of holistic well-being, there is a lack of research focused on their influence on undergraduate university students in Africa [15]. Previous studies have been conducted in high-income and Western countries within different social contexts [16]. This ultimately limits the generalisability of their findings to African settings. Furthermore, the literature has placed priority on physical health over psychosocial factors [17]. Regardless of these challenges, there is a growing recognition of the need for psychosocial research in SA, particularly to inform public health strategies and improve health outcomes among young adults, such as undergraduate university students [18]. One way of understanding PA engagement and the psychosocial factors that influence health enhancing behaviour among undergraduate university students is through creative methodologies.

### 1.2. The Use of Creative Methodologies

Creative methodologies have been considered instrumental for enhancing the understanding of embodied experiences [19]. Poetry, specifically, has increasingly been recognised as a legitimate research method, with poetic inquiry valued both as a tool for conducting research and as a meaningful outcome of the research process [20]. For example, a recent systematic review of arts-based methods used to explore young adults’ psychosocial needs and experiences found that creative approaches not only empower participants to express their personal narratives but also help reduce stigma and foster a sense of agency [21]. The review further identified mental health as the most common psychosocial issue and emphasised that creative methods enable a deeper understanding of such challenges by allowing participants to share their embodied experiences [21]. Similarly, another study focusing on health behaviours, emphasised the value of research poetics, particularly “I” poems, in health promotion by offering deeper insight into the health behaviours of individuals [22]. Their results indicated that by capturing the personal narratives in poetic form, researchers were able to explore how relationships—with family, peers and healthcare providers—shaped their health choices. This suggested that not only does this type of approach enrich the data analysis but also uncover hidden tensions and inequities, leading to more effective and empathetic health promotion strategies [22]. Thus, to fathom the experience of health behaviours among undergraduate university students, the Listening Guide serves as a powerful tool for generating I-poems, which enable the students’ voices and uncover the complex, relational dimensions of their PA engagement levels and accompanying psychosocial factors.

### 1.3. The Listening Guide Approach for the Current Study

In 1982, Carol Gilligan’s *In a Different Voice* highlighted the need to incorporate women’s voices into psychological research, particularly in developmental and moral psychology [23]. Through her studies on topics such as abortion and moral judgments, she observed that psychology was dominated by male perspectives, which were often generalised to women [23,24]. This led to the development of the feminist ‘Listening Guide’ method, which focuses on understanding the individual’s subjective experience through active listening and contextual analysis [25]. The ‘Listening Guide’ leads the focus by assessing the psychological features of the subject, how they navigate the specific terrain of inquiry and what parts of their subjective experience speaks to the researcher’s explorations. This ‘Guide’ is a way of engaging with the participant and their data through close listening, active responding and contextual positioning [26]. Where both the ‘Listening Guide’ and the I-poem were used informally in ‘*In a Different Voice*’, they have now been considered a more creative and participatory mode of inquiry [27]. The ‘Listening Guide’ and the use of I-poems, where each line starts with “I”, emerged to better capture personal voices [26,28]. I-poems are created through a flexible, systematic approach that allows for a creative, emotional engagement with data, offering qualitative insights that quantitative methods cannot [29]. This approach enables the capture of a ‘felt sense’—the emotional responses participants experience, which are conveyed in their words [24,30]. These words in the form of poetry take this ‘felt sense’ a step further, reaching parts of the human psyche that simple prose, and certainly quantitative research, cannot [20,31]. By highlighting the emotional dimensions of PA, I-poems enable a more focused analysis of how undergraduate university students’ emotions intersect with health and well-being behaviours. This ultimately sheds light on the intricate psychosocial factors that motivate or impede participation in exercise and influence a student’s holistic well-being.

Although the use of creative arts-based methods has become a novel research method within qualitative research, limited evidence exists on how these methods have been employed to understand undergraduate university students’ PA participation and the psychosocial factors that shape their engagement experiences. Previous studies have either focused on (1) young people’s emotions during COVID-19 [27], (2) disabled cyclists [32], or (3) adventurous play among children [33]. Furthermore, few have studied the health behaviours of university students through poetic inquiry [21,34]. Thus, a gap in the research exists regarding the application of creative arts-based methods to explore undergraduate university students’ PA participation and the psychosocial factors influencing their engagement, particularly in the context of university settings [18]. Therefore, through the use of the ‘Listening Guide’, this study aimed to explore students’ PA participation by providing insights into the psychosocial factors that shape their experiences.

## 2. Materials and Methods

### 2.1. Study Design and Location

This study qualitatively explored students’ PA participation, by providing insights into the psychosocial factors that shape their experiences. This study was conducted among students at a University in the Western Cape province of SA. Online interviews via Google Meet were used to ensure that all students, regardless of whether they were on or off campus, had an equal opportunity to participate in this research study. This enabled the researcher to reach a broader and more diverse student group.

### 2.2. Sampling and Recruitment

This study was part of a broader mixed-methods research project investigating the relationship between psychosocial factors and PA participation [9]. For the purpose of this study, only the qualitative findings are presented. Eighteen undergraduate students (*n* = 18) were conveniently sampled after completing a quantitative survey in the larger study and voluntarily consented to participate in online interviews. Eligibility criteria required participants to be full-time registered university students at a university situated in the Western Cape province of SA. Students were excluded if they were under 18 years of age, registered for part-time or semester-based courses or if they declined to provide written informed consent. Prior to data collection, all participants received an information sheet outlining the study’s objectives, potential risks and benefits and the researcher’s contact information.

### 2.3. Interview Instrument

The semi-structured interview questions were formulated based on a review of the relevant literature [5,8,11] and findings from a previous study that adopted a mixed-methods approach [9]. The primary researcher compiled the semi-structured interview schedule (Table 1), which included questions concerning PA.

### 2.4. Data Collection

Individual interviews were conducted online in English, the primary language of instruction at the institution. Prior to the main data collection, a pilot study was carried out in July 2023 with two undergraduate students who were not part of the final sample. The pilot study aimed to assess the duration of the interviews and test the research methods [35]. To preserve the integrity of the main study’s findings, data from the pilot participants were excluded from the analysis. From July to August 2023, a total of 18 interviews were conducted via the Google Meet platform, with each session lasting between 45 and 60 min. Interviews were audio-recorded with the consent of the participants. The interview process followed a structured format, including introduction and rapport building, questioning, probing, summarisation, and concluding remarks. Participants were asked open-ended questions concerning PA and its influencing psychosocial factors (such as ‘What does PA mean to you? What influences your engagement in PA?’). Saturation was reached after the 18th participant, as no new themes or insights emerged from the data. Data were analysed (verbatim) immediately after each interview. The interview transcripts were transcribed by the primary researcher and subsequently sent to participants for member checking. This provided participants with the opportunity to review and supplement any missing or overlooked details. Participants were asked to confirm the accuracy of the transcripts and the information provided. Data analysis was initiated once the member checking process was completed (where participants were provided with their transcripts to review and verify the accuracy of their information, allowing them to offer corrections or clarifications as part of the member checking process).

### 2.5. Data Analysis Steps in ‘The Listening Guide’ Approach of Qualitative Research

**Figure 1:**
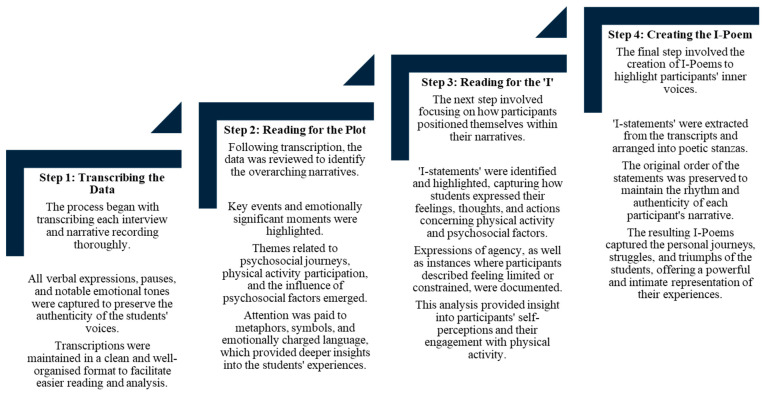
Data analysis steps in ‘The Listening Guide’ approach of qualitative research.

A reflexive thematic analysis approach was adopted for this current study [36]. An inductive approach was employed, ensuring that the analysis was driven entirely by the data, instead of being guided by existing theories or frameworks [37]. The transcribed interview recordings were uploaded to the qualitative analysis software, namely Atlas Ti., V8. To ensure anonymity, the participants’ names were replaced with pseudonyms. Moreover, previous research employing the same Listening Guide approach for qualitative studies was adapted and utilised [30] (Figure 1). Due to the anonymised and online nature of the current study and the large number of participants, the process of each step was adapted to suit these circumstances. The ‘listenings’ were instead ‘readings’, and active responding was not possible [30]. This approach involved four steps. Step 1 focused on transcribing the data. In this study, all participants’ interviews were transcribed verbatim to ensure that all verbal expressions, pauses and notable emotional tones were captured to preserve the authenticity of the students’ voices. Transcriptions were maintained in a clean and well-organised format to facilitate easier reading and analysis. Step 2 focused on reading for the plot. In this stage, researchers asked the questions, ‘What is the story that is being told?’, ‘What are the students’ experiences?’ Key events and emotionally significant moments were highlighted. Themes related to PA participation and the influence of psychosocial factors emerged. Attention was paid to metaphors, symbols and emotionally charged language, which provided deeper insights into the students’ experiences. Step 3 focused on reading for the ‘I’; this included how participants positioned themselves within their narratives. ‘I-statements’ from the transcriptions were identified and highlighted, capturing how students expressed their feelings, thoughts and actions concerning PA engagement and their psychosocial factors. Expressions of agency, as well as instances where participants described feeling limited or constrained, were documented. This analysis provided insight into participants’ self-perceptions and their engagement with PA. Lastly, Step 4 focused on creating the ‘I’ poem, generated from the transcriptions, to highlight participants’ inner voices. The ‘I-statements’ were extracted from the transcripts and arranged into poetic stanzas. The original order of the statements was preserved to maintain the rhythm and authenticity of each participant’s narrative. The resulting I-poems captured the personal journeys, struggles and triumphs of the students, offering an intimate representation of their PA and psychosocial experiences.

Furthermore, the I-poems were thematically analysed. Thematic analysis was selected due to its effectiveness in identifying patterns across the data, reflecting the participants’ lived experiences and perspectives [37]. This thematic process followed the four phases of theme development, namely initialisation; construction; rectification; and finalisation [38].

In the initialisation phase, the transcriptions were carefully reviewed, with meaning units highlighted and codes generated, alongside the recording of reflective notes (for instance, MH for mental health, PA for physical activity: for example, “I feel anxious when I skip the gym” was coded as MH(A) for anxiety, and “I lose motivation when I’m alone” as M(L) for loneliness influencing motivation). In this phase, the Listening Guide was applied, which focuses on understanding the participants’ voices and perspectives through a nuanced, multi-layered analysis. Each participant’s narrative was considered not only for content but for tone, emotion and emphasis, capturing the depth of their experience. In the construction phase, the focus shifted to categorising the data by comparing the codes for similarities and differences relevant to the research questions, and assigning labels that accurately represented the participants’ perspectives. The Listening Guide approach further supported this process by examining the participants’ varying layers of meaning, such as their affective and relational expressions, helping to ensure the themes were grounded in the lived experiences of the participants. The codes were then grouped into themes, reflecting both the content and emotional undertones present in the data (for instance, MH(A) for anxiety and MH(D) for depression were grouped under the main theme: Mental health and the subtheme “Mental health challenges”). During the rectification phase, a self-correcting process was applied, where the researchers distanced themselves from the data to ensure consistency between the methodology and the findings. This phase included revisiting the Listening Guide’s framework to reassess the interpretations and ensure the themes remained true to the participants’ voices. This iterative process enabled the researchers to navigate between the data and the study’s objectives, ensuring alignment and coherence while respecting the complexity of the participants’ experiences. Once the themes were established and cross-referenced with the literature, thematic statements were formulated. Throughout this process, the Listening Guide was used to ensure that the themes reflected not only the content but also the emotional and relational aspects of the participants’ narratives. This approach allowed the researchers to capture the full complexity of the themes, ensuring that the subtleties of tone, emotion and emphasis were integrated into the thematic statements. In the finalisation phase, the researchers developed a narrative that connected the themes to address the study’s objectives. This narrative was informed by the Listening Guide, ensuring that the final interpretation honoured the participants’ voices and perspectives. Subsequently, the themes were organised into a thematic table, which was reviewed and revised by the co-researchers. Sub-themes were merged where similar contexts or emotional undertones were shared, ensuring coherence across the findings. The table underwent multiple revisions, incorporating the feedback and refining the connections between themes, before the final main themes and sub-themes were agreed upon by the researchers [9].

I-poems were chosen as an analytical tool to complement the traditional thematic analysis by centring students’ voices in a more personal, affective and embodied way [30]. While thematic analysis effectively identifies patterns across data, it may sometimes distance the reader from the emotional depth and individuality of participants’ experiences. I-poems preserve the participants’ language, rhythm and emotional cadence [29]. This method is inherently reflective, offering participants the opportunity to explore their thoughts and emotions surrounding their PA experiences. Through this reflective process, students are able to express not only their successes but also the internal struggles they may face [29], such as battling with motivation or coping with social pressures that influence their commitment to exercise. It is through I-poems that these complex emotional layers come to the surface, allowing participants to articulate moments of self-doubt, joy, frustration or accomplishment. These reflections provide a more holistic view of health behaviours, one that acknowledges both the highs and the lows that form the foundation of an individual’s PA journey. The poetic form amplifies the affective dimensions of their psychosocial journeys, such as vulnerability, resilience and self-discovery, that might be diluted in traditional coding processes. Traditional research methods, such as surveys, often focus on broad measures that may overlook these emotional cues [34,39]. Therefore, by humanising the data and making student voices more audible and impactful, I-poems provided an additional interpretive layer that enriched both the analysis and the practical applications of the findings [29].

### 2.6. Reflexivity and Trustworthiness

Reflexivity and trustworthiness are essential in qualitative research to ensure objectivity and credibility. Reflexivity requires researchers to reflect on how their personal assumptions may influence the research process [40]. In this study, the researchers examined their biases and consulted with co-researchers for peer review, ensuring objectivity and reporting in the third person. Trustworthiness may be established through credibility, transferability, and confirmability [41,42]. Credibility was ensured through triangulation and member checking, while transferability was achieved through thick description to make findings applicable to similar populations. Confirmability was maintained by being transparent about biases and documenting decisions in audit notes [43].

### 2.7. Ethical Considerations

Ethics approval for this study was obtained from the Humanities and Social Sciences Research Ethics Committee at the University of the Western Cape (Reference: HS21/10/24) prior to its commencement. Permission was also granted by the Registrar’s Office to recruit students for participation. Participation in this study was entirely voluntary, and students who agreed provided electronic informed consent. Following consent, the researcher and participants arranged interviews at mutually convenient dates, times and locations. Participants had the right to withdraw from this study at any time without any consequences and were informed that they could skip any questions they preferred not to answer. This study adhered to the principle of non-maleficence, ensuring that no harm was caused during the interview process. To maintain confidentiality and anonymity, pseudonyms were used in place of participants’ real names.

## 3. Results

### 3.1. Demographics

Table 2 shows the demographic information of the students who participated in this study. The majority of participants were female (9 out of 11), with ages ranging from 19 to 23 years. This study spans across various academic disciplines, including Sport, Recreation and Exercise Science, Physiotherapy, English and Sociology, Social Work, Biotechnology, Nursing, Industrial Psychology, and Sociology. Most participants were in their second (*n* = 4) or third (*n* = 4) year of study, with one participant in their first year and two in their fourth year.

### 3.2. Thematic Categories and I-Poems

Table 3 presents the main and sub-thematic categories derived from the participants’ narratives. Four key themes, PA, mental health, motivation and social support, were identified, each with four corresponding subthemes. An I-poem was created for each of the 18 undergraduate university students in this study. However, for the purpose of this research article, only 11 I-poems were included. This was due to the need to focus on a manageable subset of data that most effectively represented the key themes and experiences relevant to the study’s objectives. The selected 11 participants were chosen based on the diversity of their responses, ensuring that a broad range of perspectives on PA and its influence on psychosocial factors were captured. Mental health as a psychosocial factor was a prominent topic among the student participants, which ultimately determined their health behaviours. The participants’ I-poems spoke about mental health, such as anxiety, depression and stress and its role on determining their health behaviours.

**Figure 2:**
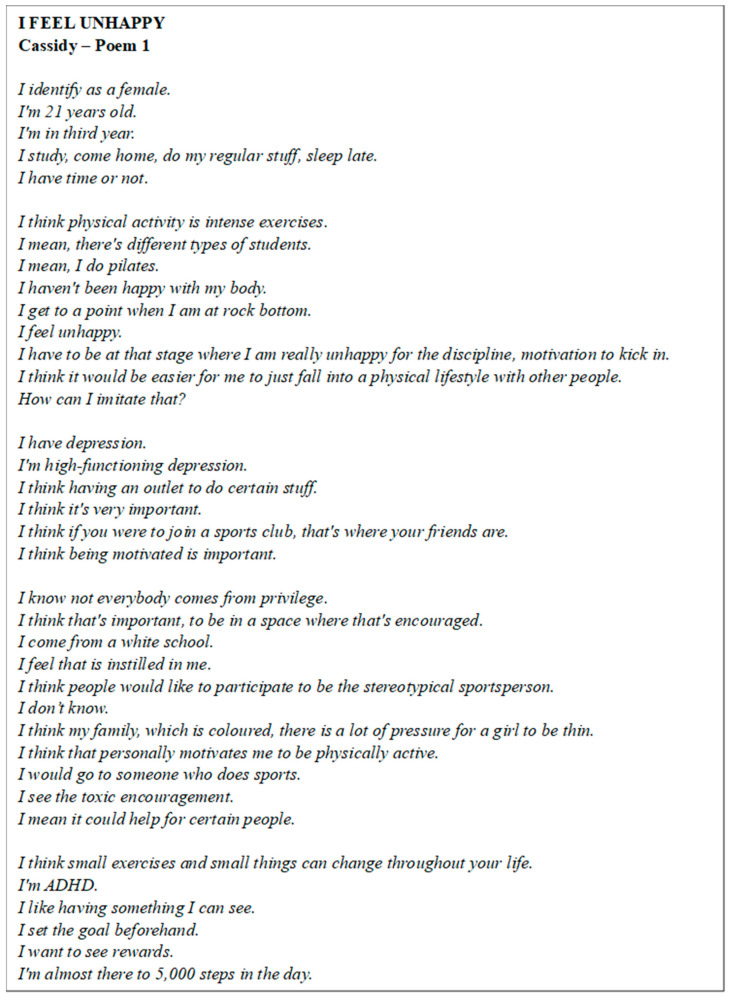
Poem 1.

Poem 1 shows Cassidy’s view (Figure 2). The participant reflected on their engagement with PA, describing it primarily as intense exercise, although they also participated in Pilates and set smaller, achievable goals such as reaching 5000 steps per day. They acknowledged the connection between PA and mental health, noting that it served as a crucial outlet for managing high-functioning depression and attention-deficit/hyperactivity disorder (ADHD). She emphasised the importance of social support in motivating PA, expressing that being part of a sports club would provide a space for both social connection and motivation. Family dynamics and societal pressures, particularly regarding body image, also played a significant role in their motivation to stay active, although they recognised the potential for toxic encouragement within these influences.

**Figure 3:**
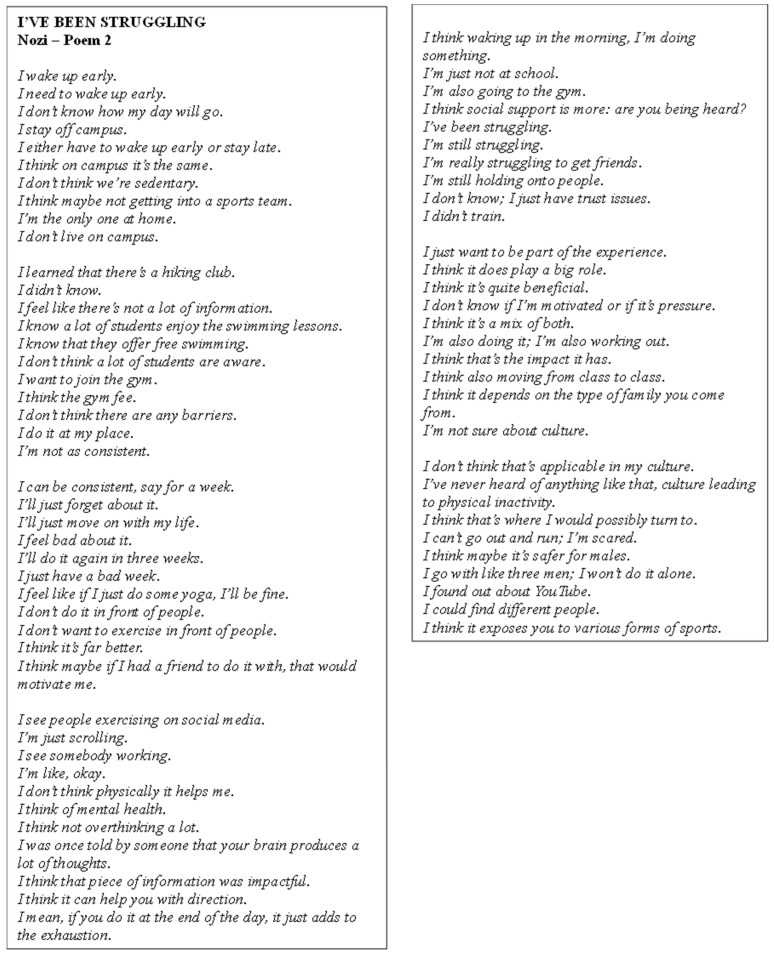
Poem 2.

Poem 2 showcases Nozi’s perceptions (Figure 3). She reflected on her daily routine, acknowledging the challenges she faced in balancing PA with academic life while living off-campus. They expressed interest in physical activities such as yoga and joining the gym, but struggled with consistency, often finding it difficult to maintain regular exercise. Nozi highlighted the role of social support, suggesting that having a friend to exercise with would increase their motivation. They mentioned being influenced by social media but felt that PA did not necessarily help them physically; instead, it benefited their mental health by reducing overthinking. Nozi recognised the importance of mental health and the impact of exercise on their well-being, though they also felt isolated in terms of social connections, citing trust issues and difficulty in making friends. Cultural factors were mentioned as a potential barrier to PA, but Nozi expressed interest in exploring different sports through platforms like YouTube.

**Figure 4:**
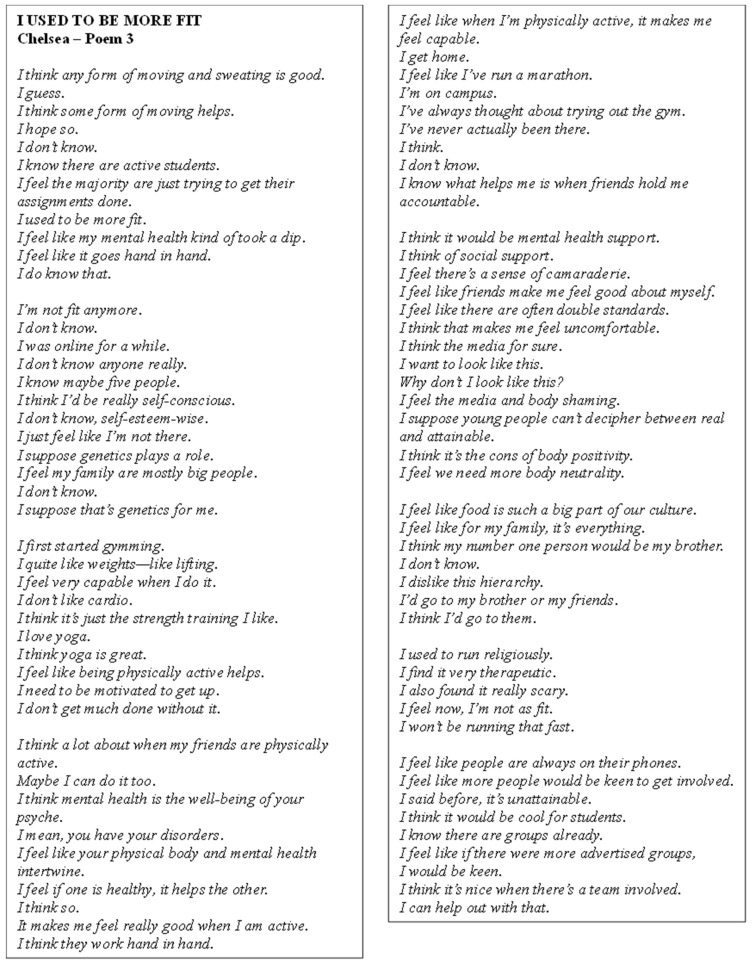
Poem 3.

Poem 3 delves into Chelsea’s experiences (Figure 4). The participant reflected on their past engagement with PA, acknowledging the importance of moving and sweating for overall well-being, though they struggled with consistency due to a decline in their fitness and mental health. They expressed a sense of self-consciousness and low self-esteem, especially regarding body image, which was influenced by family genetics and social comparisons with media portrayals. Chelsea highlighted the connection between mental health and PA, noting that exercise, particularly strength training and yoga, improved their sense of capability and well-being. The participant mentioned social support, recognising the positive influence of friends, holding them accountable and providing camaraderie, which motivated them to engage in PA. Despite this, they expressed concerns about body image issues, body shaming and the unrealistic portrayals in the media, calling for more body neutrality. They also suggested that better advertising of group activities and sports could encourage more students to get involved.

**Figure 5:**
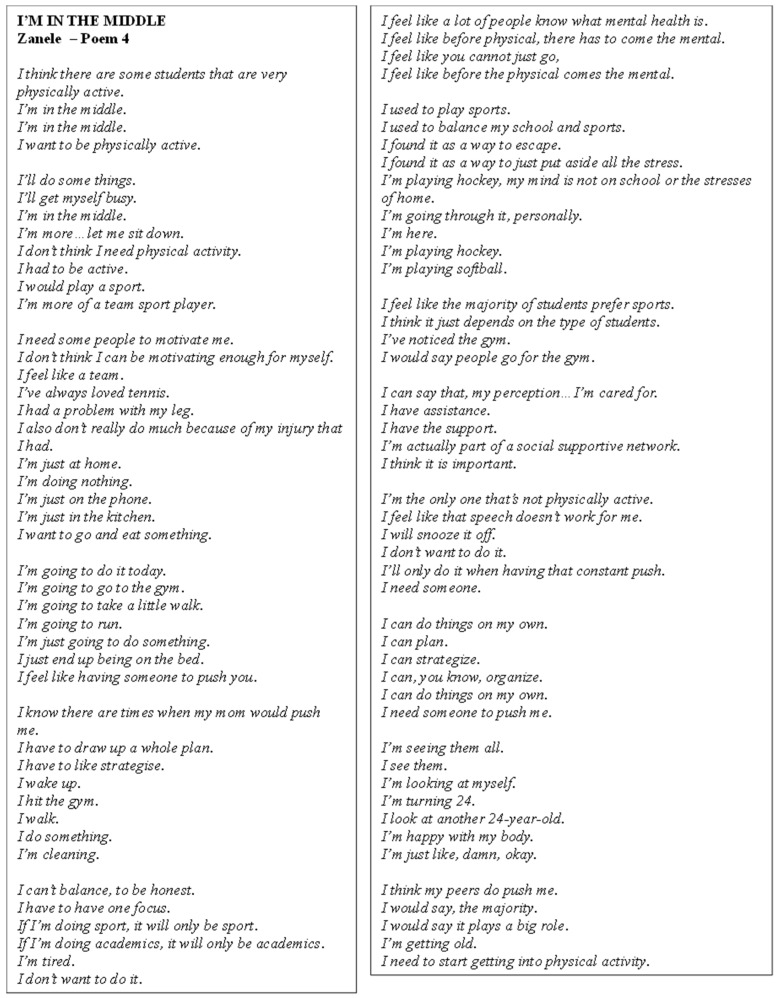

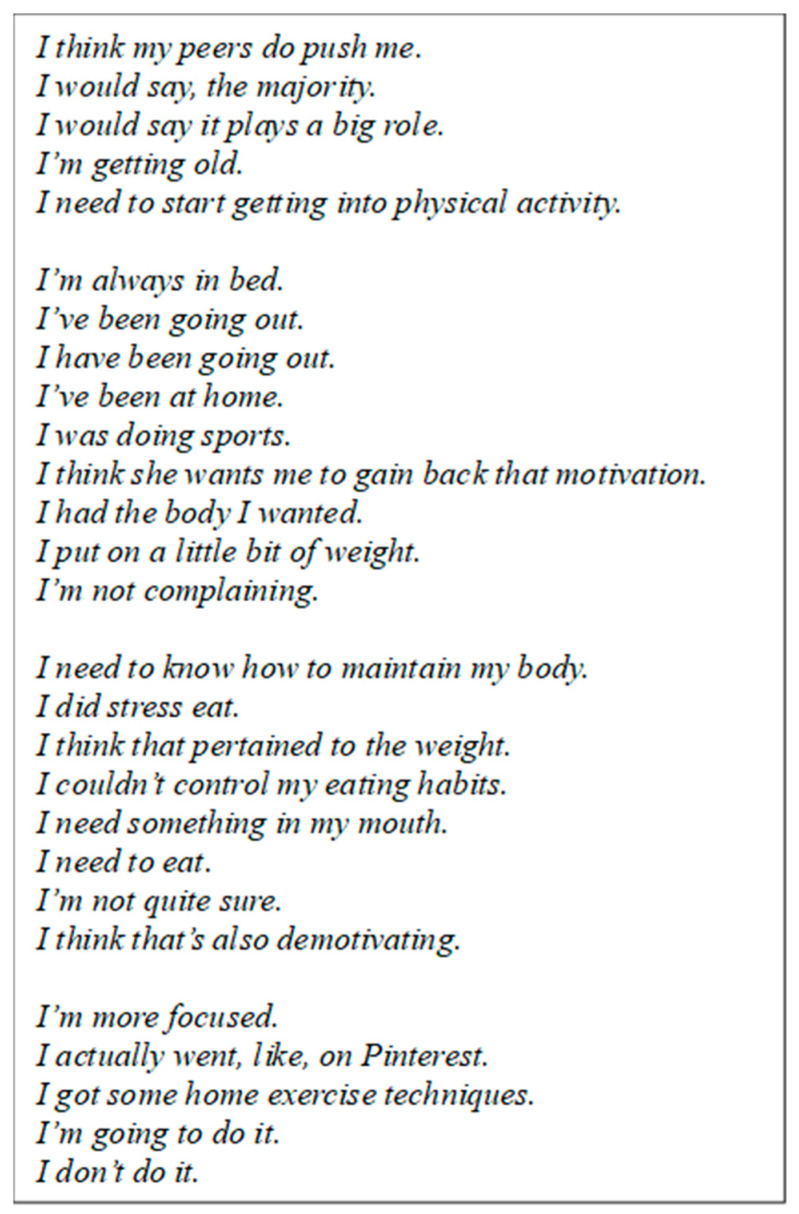
Poem 4.

Poem 4 shows Zanele’s journey (Figure 5). She expressed a desire to be physically active, though they felt they were currently in the middle, neither highly active nor sedentary. They reflected on the importance of mental health before PA, emphasising that mental well-being was foundational to engaging in exercise. Zanele shared past experiences in team sports such as hockey and softball, which served as a form of escape from academic and personal stresses. They acknowledged the social support they received from peers, which played a crucial role in their motivation to engage in PA, though they often felt that they needed an external push and accountability to stay active. Despite recognising their own ability to plan and organise, the participant admitted that they struggled to maintain motivation without others to encourage them. They also mentioned the impact of body image, comparing themselves to peers and feeling pressure as they approached their mid-20s. Their PA was further hindered by an injury, which had contributed to a more sedentary lifestyle. The participant stressed the importance of external motivation, especially from family or a social network, in maintaining an active routine. Although they expressed frustration with balancing academics and sports, they recognised that having a plan and being pushed by others, such as their mother, helped them stay on track with their fitness goals.

**Figure 6:**
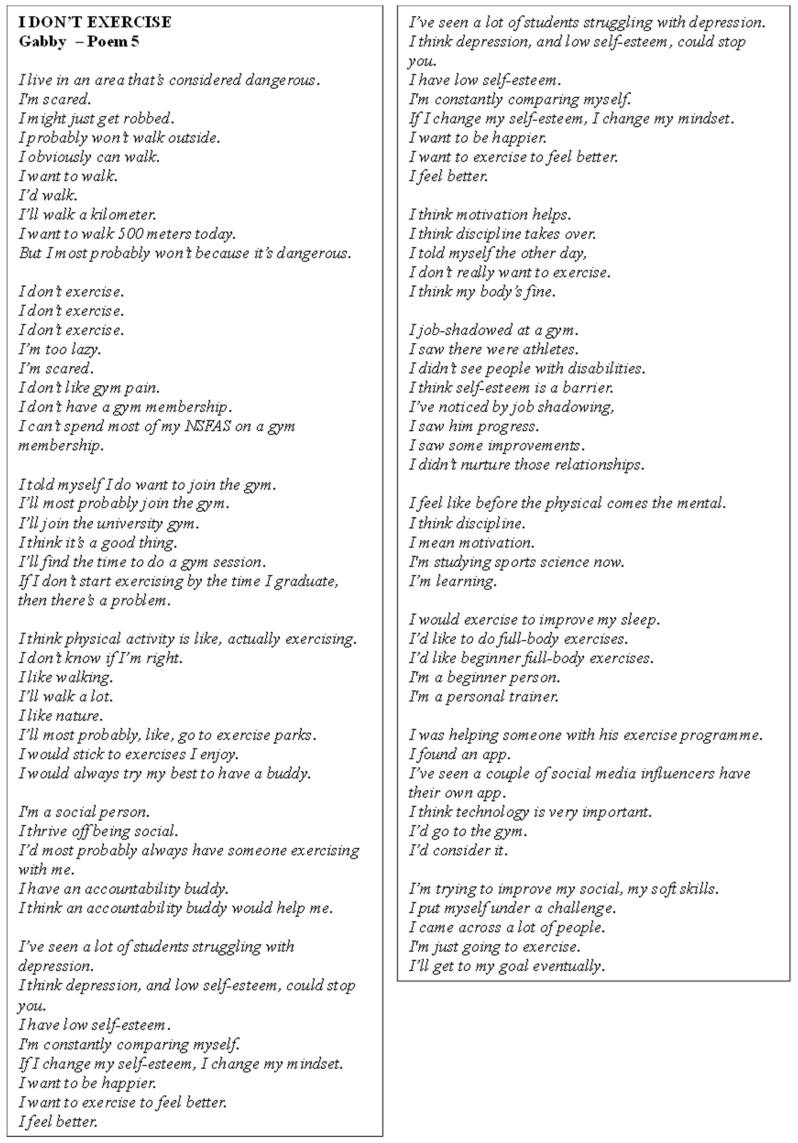
Poem 5.

Poem 5 focuses on the reflections of Gabby (Figure 6). She expressed concern about living in a dangerous area, which prevented outdoor activity. She mentioned struggling with low self-esteem and depression, believing these factors hindered motivation for exercise. Gabby wanted to improve her mindset and felt that better mental health would lead to better motivation. While she recognised the importance of motivation and discipline, she often felt too lazy or scared to exercise, citing financial constraints like not being able to afford a gym membership. She was interested in beginner exercises, particularly walking and nature activities, and preferred exercises she enjoyed. Gabby also valued social support and thought an accountability buddy would help. She was studying sports science and planned to join a university gym if she didn’t start exercising before graduation, hoping to improve her physical and mental health.

**Figure 7:**
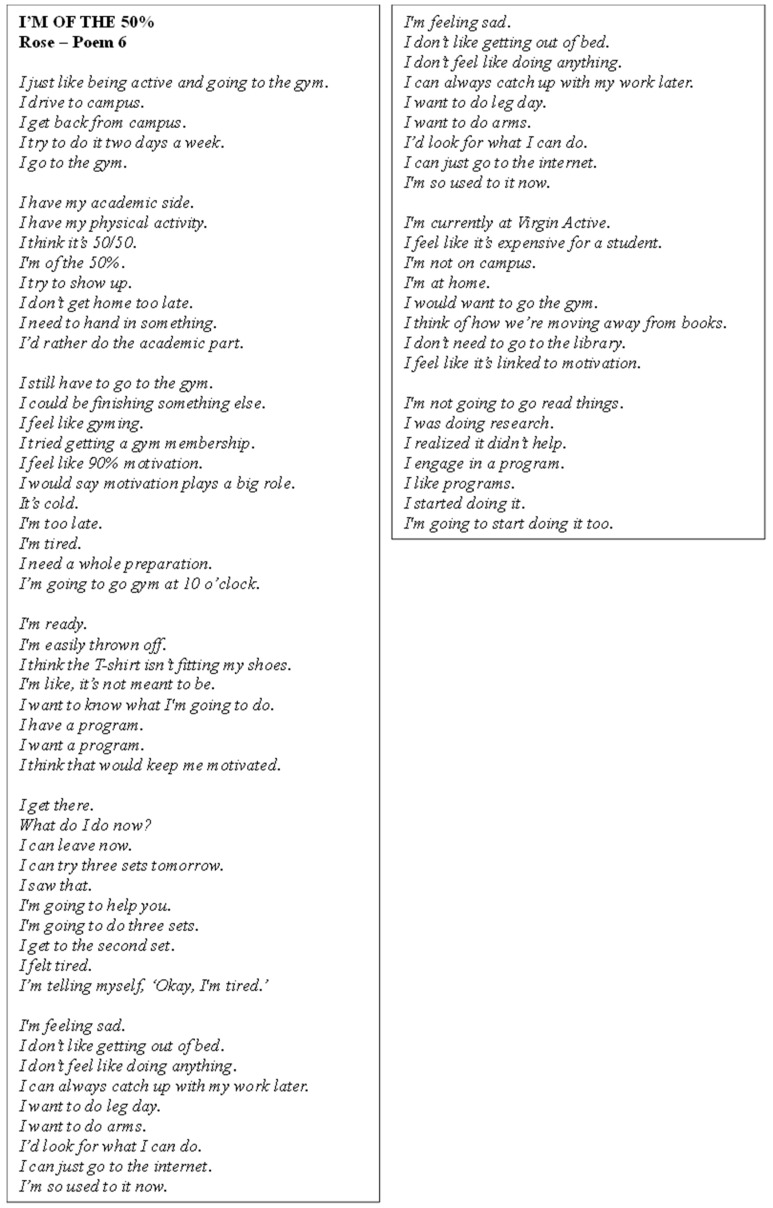
Poem 6.

Poem 6 delves into Rose’s experiences (Figure 7). She communicated a desire to stay physically active, particularly through gym workouts, but faced challenges with motivation, especially when feeling sad or tired. Rose acknowledged the importance of motivation in her routine, often relying on external sources like programs and the internet to stay engaged. Financial constraints, such as the cost of a gym membership and time management between academic work and PA also posed barriers, but she aimed for a balance between the two. Social support appeared to play a minor role, as she sometimes relied on others at the gym for encouragement. Despite setbacks like lack of energy or distractions, Rose continued to strive to incorporate PA into her routine, recognising it as an essential part of her well-being.

**Figure 8:**
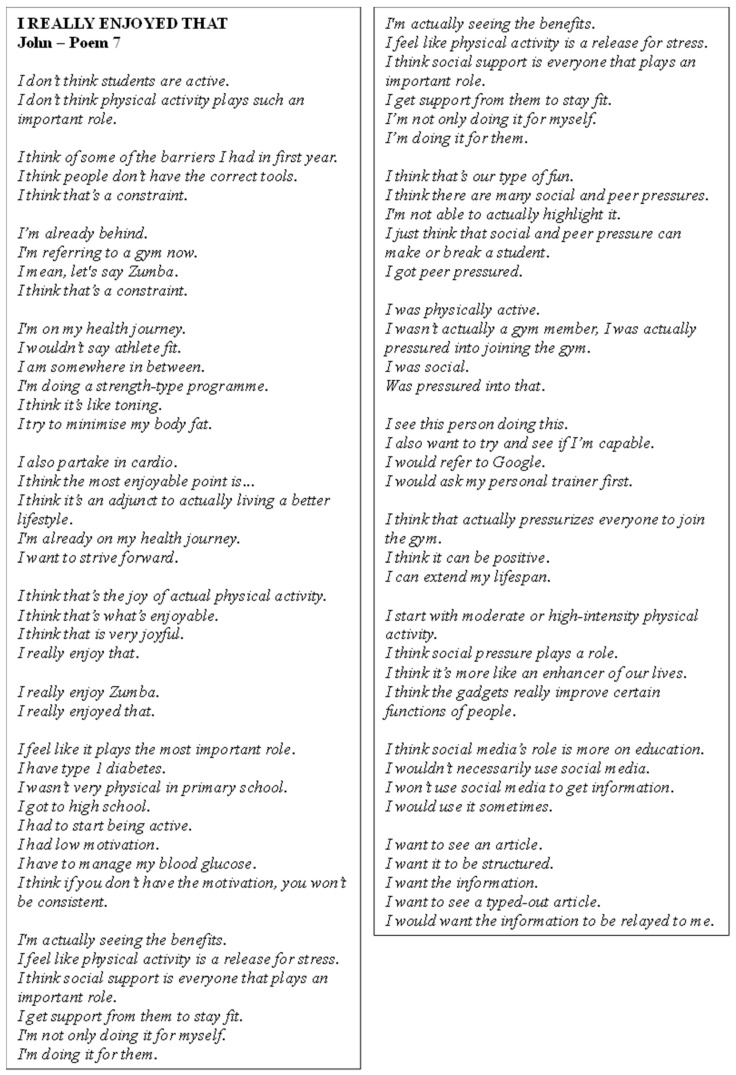
Poem 7.

Poem 7 shows John’s perspectives (Figure 8). He reflected on his evolving relationship with PA, initially sceptical about its importance, but later recognising its benefits for stress relief and overall well-being. John acknowledged the crucial role of social support, both from peers and trainers, in staying active and motivated, emphasising that he was motivated not just for himself but also for others. Peer pressure and social pressure influenced his participation in activities such as joining the gym, though he viewed these pressures as both positive and negative. Despite challenges such as low motivation and health constraints related to type 1 diabetes, he remained committed to his health journey, engaging in activities like Zumba and strength training. John also noted the importance of having the right tools and information, sometimes seeking resources like articles for guidance. His experience suggested that motivation, social support, and peer influence were key factors in sustaining an active lifestyle.

**Figure 9:**
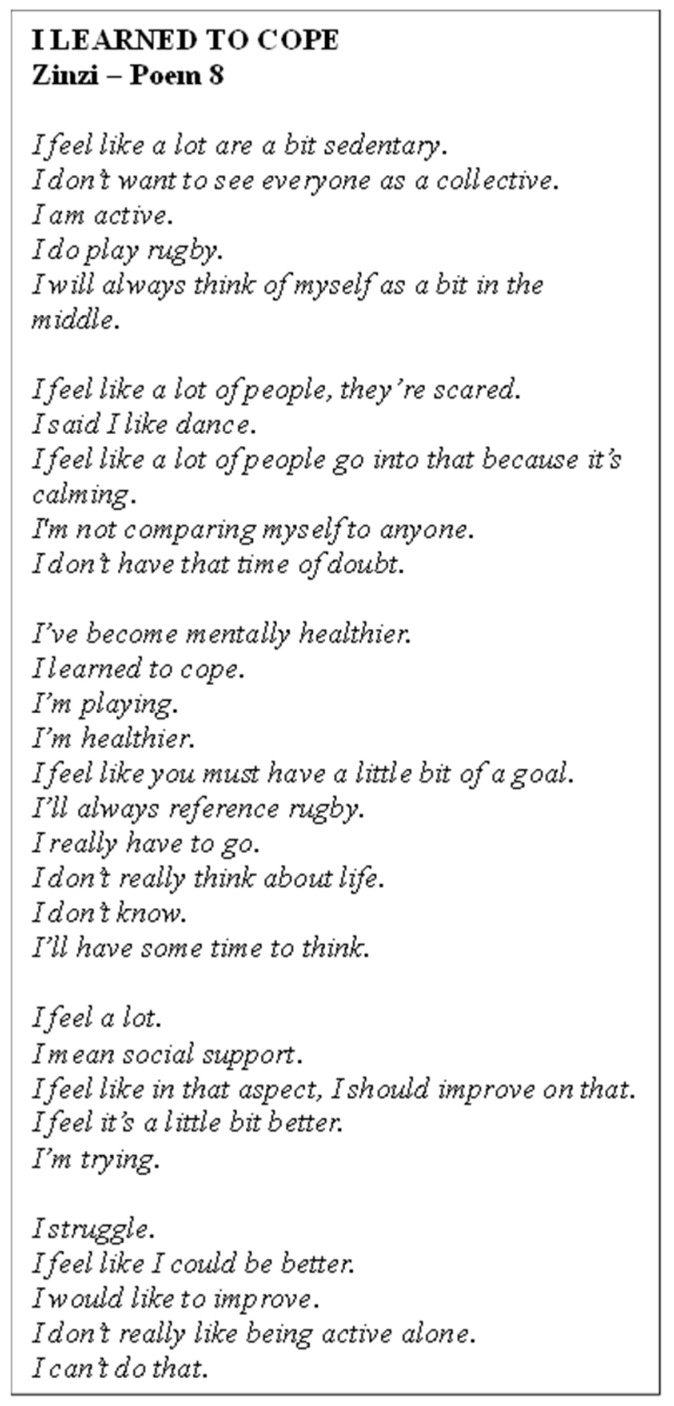
Poem 8.

Poem 8 delves into Zinzi’s journey (Figure 9). She discussed her experience with PA, identifying as active through rugby but also feeling that many people were sedentary. Zinzi highlighted the calming effects of dance and the importance of having a goal to stay motivated. She shared how PA had contributed to her mental health, describing herself as feeling mentally healthier and better able to cope. Zinzi expressed a desire for social support, acknowledging that she could improve in this area and preferred not to be active alone. Motivation played a key role in her engagement, with rugby serving as a consistent reference point for her goals, although she also expressed challenges in staying fully motivated and improving.

**Figure 10:**
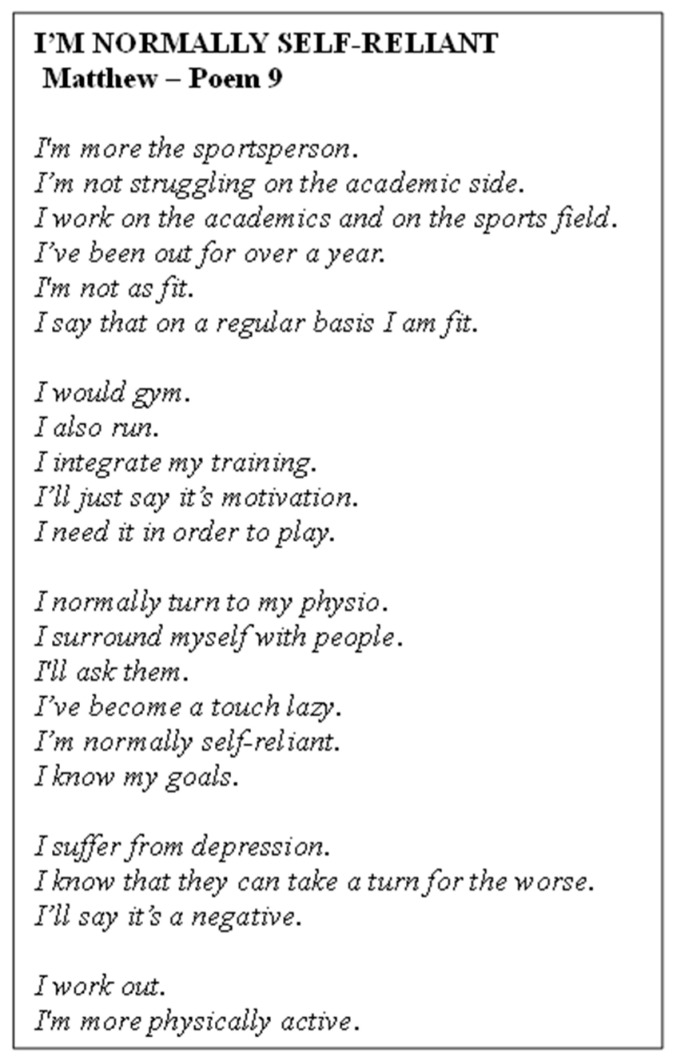
Poem 9.

Poem 9 shows the perspectives gathered from Matthew (Figure 10). He reflected on his PA journey, noting that while he had once enjoyed going to the gym, his motivation declined due to financial challenges and a lack of stability, leading to a decrease in activity. Matthew expressed a desire to return to the gym, recognising the positive effects exercise had on his mental health, such as feeling calmer and more focused. Despite currently being less active, he had planned to resume exercising and was motivated by the prospect of improving his well-being. The participant emphasised the importance of having social support for staying active, though he remained uncertain about how external factors like religion influenced his PA. He relied on self-directed learning through resources such as Google and YouTube to inform his fitness journey, valuing the convenience and accessibility of online information.

**Figure 11:**
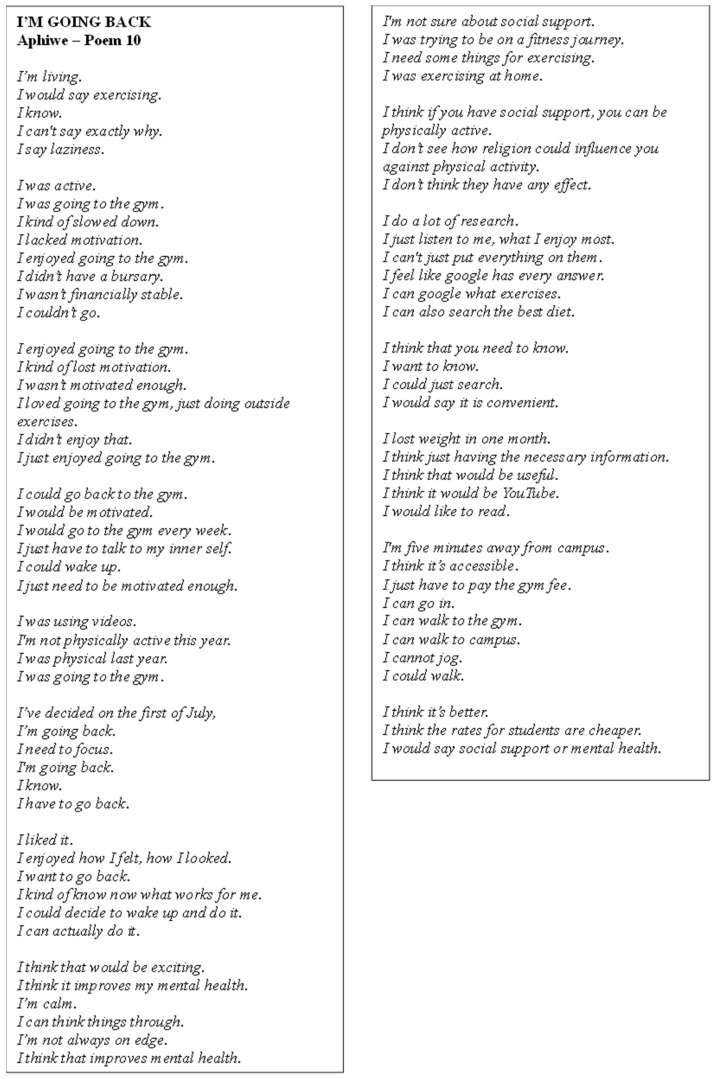
Poem 10.

Poem 10 (Figure 11) focuses on Aphiwe’s experiences as a sportsperson, balancing academic and PA commitments. She acknowledged a decline in fitness after being out for over a year but still viewed herself as generally fit. The participant integrated her training through the gym and running, motivated by the need to stay fit for sports. She highlighted the importance of motivation in maintaining her PA routine. While she typically relied on self-reliance, she also sought social support from a physiotherapist and peers when needed. Aphiwe was open about her struggles with depression, recognising its potential to worsen, but remained committed to working out and staying active to improve her mental health.

**Figure 12:**
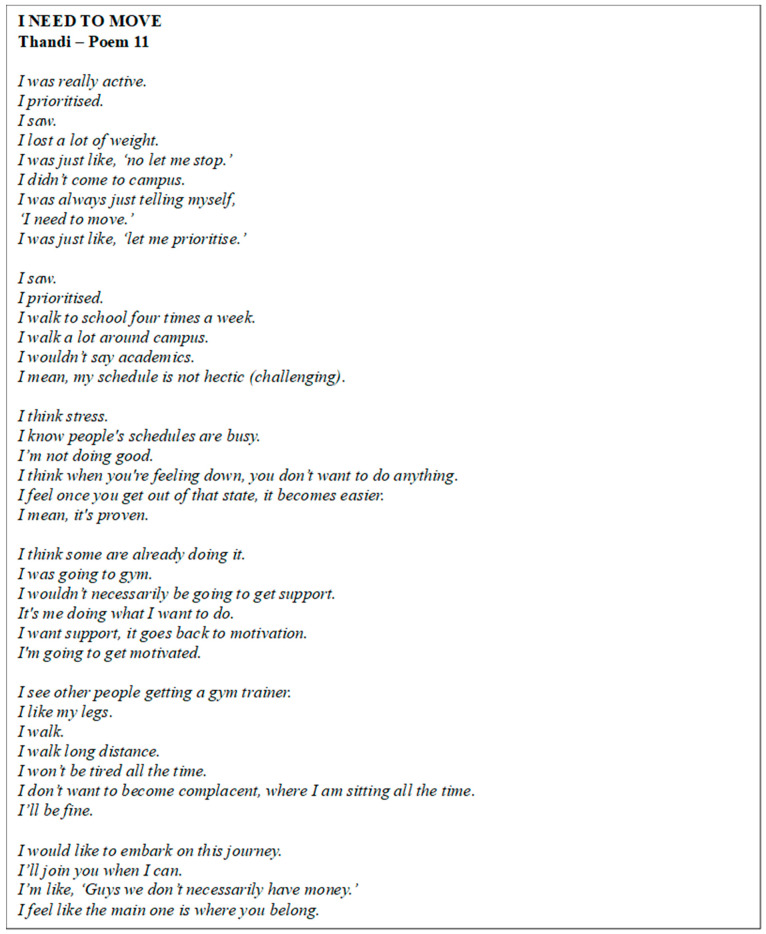
Poem 11.

Poem 11 shows Thandi’s perspective (Figure 12). The participant expressed a fluctuating relationship with PA, noting that she had previously enjoyed exercising, particularly at the gym, but had recently struggled with motivation due to financial constraints and a lack of stability. She highlighted the importance of social support and mental health in staying active, believing that having access to support could enhance PA engagement. Thandi had used resources like Google and YouTube for fitness information, showing a preference for self-reliance in learning and motivation. She reflected on the positive impact of exercise on her mental health, feeling calmer and more able to think clearly when active. Despite current challenges, Thandi was determined to return to the gym, motivated by the positive effects it had had on her physical and mental well-being in the past.

**Figure 13:**
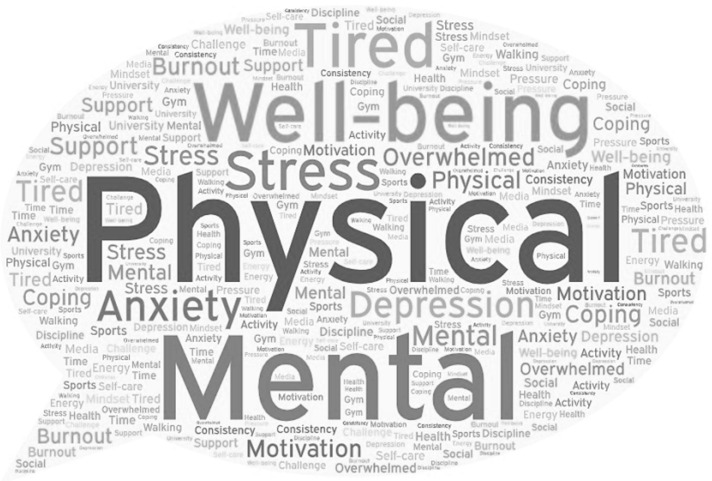
Common keywords derived from the I-poems.

Based on the findings derived from the I-poems, a word cloud was generated [using WordArt.com, 2009 (accessed on 12 February 2025)] (Figure 13) in order to show the prominent words that were generated across the 11 I-poems.

## 4. Discussion

This study aimed to explore undergraduate university students’ PA participation by providing insights into the psychosocial factors that shaped their experiences. The I-poems provided a unique way for undergraduate university students to articulate their personal journey with PA, which ultimately captured their subjective experiences and emotional responses. Based on the findings derived from this study, four major themes were generated, namely, PA and students’ perceptions thereof, the influence of mental health, the role of social support and motivation as a source of encouragement. Psychosocial factors such as mental health, motivation and social support have been classified as determinants of health by various researchers [14,44] and either promote or hinder regular PA levels [10]. Therefore, these psychosocial factors must be subjectively understood in order to enhance PA health behaviours among undergraduate university students.

### 4.1. Physical Activity

Physical activity (PA) has been proven to be beneficial in several ways, namely, physical health, psychological well-being, body image and enhanced quality of life [45,46]. Participants from this study expressed diverse experiences with PA, with many engaging in activities such as gym workouts, sports [like rugby] and dance. They noted that these activities contributed positively to their physical well-being, helping them feel healthier, more energetic, and capable of managing stress (*n* = 7 out of 11, 64%). Aligned with previous research, PA has been proven to be beneficial for holistic well-being, including decreased obesity, reduced hypertension, improved mental health, greater support networks and a heightened sense of motivation [47,48,49]. Nevertheless, PA participation among young people is not sufficient to maintain an active lifestyle [5] as research indicated that students are not meeting the recommended PA guidelines [50]. Based on the findings derived from this study, students provided plausible reasons for this decline in PA. Some mentioned PA barriers such as external factors like financial instability and the lack of time, particularly when they were unable to afford gym memberships or were overwhelmed with the academic workload. Despite these challenges, many participants showed a desire to return to more regular exercise, with some even setting clear goals, such as going back to the gym or engaging in home workouts. This suggests that students recognise the benefits of a healthy lifestyle and are willing to take proactive steps to enhance their holistic well-being (with an emphasis on their mental health as a starting point).

### 4.2. Mental Health

Mental health emerged as a dominant theme across all the I-poems, as students consistently linked their PA levels to their emotional and psychological well-being. Students expressed feelings of stress, anxiety and exhaustion, which influenced their engagement in health-enhancing activities, while others struggled with consistency due to a lack of mental energy and motivation (*n* = 9 out of 11, 82%). However, the I-poems revealed a strong self-awareness among students regarding the link between their mental and physical health. Previous studies have indicated that positive mental health, characterised by lower levels of depression, anxiety and stress, has been researched as enhancing PA engagement, as individuals have more energy and desire to be physically active [51,52]. Similarly, this study found that the link between PA and mental health was frequently highlighted, with participants reporting improvements in their mood, stress levels and overall mental well-being due to engaging in physical activities. Regular exercise was seen as a means of alleviating stress, improving clarity of thought, and reducing feelings of being on edge. For some, the act of exercising provided a mental break from academic or personal pressures, allowing them to clear their mind and regain focus. In alignment with additional authors, PA has been regarded as a coping mechanism to delay the onset of mental health challenges [10]. Despite these benefits, mental health challenges as cited in this current study, such as feelings of isolation, fear of judgment, laziness, anxiety or depression, were also mentioned, with some participants noting that these obstacles hindered their ability to stay consistent with their PA routines. These results aligned with previous authors who reported that mental health disorders account for over 12% of the global disease burden, where depression affects 322 million people globally, representing 4.4% of the population and anxiety impacts approximately 260 million individuals or 3.6% of the global population [53]. Thus, poor mental health can deter individuals from participating in PA, creating a negative cycle of well-being and demotivation [54,55]. Additionally, this study found that maintaining a structured health routine was seen as important for both mental stability and physical well-being, but many found it difficult to balance academic responsibilities, social life and self-care. These results align with previous studies, which reported that academic pressure is a major source of stress, consequently discouraging university students from being physically active [56,57]. The I-poems revealed a strong self-awareness among students regarding the link between their mental and physical health. Therefore, mental health as a prominent psychosocial factor should be considered when developing student-tailored interventions [10,58]. However, mental health is not a standalone psychosocial factor that determines health behaviour. Additional aspects such as social support and motivation have been considered as intertwined elements that determine whether a student will engage in health-enhancing activities [10].

### 4.3. Social Support

Numerous studies have considered social support as a social determinant of human behaviour, particularly engagement in leisure-time PA among university students [59,60]. Social support networks such as family and peers have been regarded as PA facilitators based on their ability to provide encouragement, accountability and a sense of belonging [61]. Participants (*n* = 9 out of 11, 82%) in this study cited that social support played a significant role in their ability to remain engaged in PA. Many participants highlighted the importance of friends, family or peers in encouraging them to stay active, with some explicitly stating that they were motivated to exercise not just for themselves but for the sake of their support networks. Peer pressure was also mentioned, both as a positive motivator and as a source of pressure to conform to certain fitness activities, such as joining a gym. While some participants (*n* = 4 out of 11, 36.3%) expressed uncertainty about the influence of social support on their PA, others acknowledged (*n* = 8, 73%) that it was a crucial factor in maintaining motivation and consistency in their fitness journey. The importance of receiving social support from interpersonal relationships has been well-documented in research [62,63]. Specifically, researchers have investigated the impact of social support on the PA behaviours of individuals and reported that individuals with higher social support had higher mean PA scores compared to individuals with lower support networks [64,65]. Similarly, previous studies have reported that exercising with others was found to increase students’ sense of belonging, accountability, enjoyment and motivation [56]. Additionally, receiving social support and encouragement from others to be physically active played a significant role in shaping students’ values towards PA and enhancing their motivation [56]. This highlights the importance of social support structures for enhancing PA participation and having a positive influence on psychosocial well-being.

### 4.4. Motivation

Motivation, as a psychosocial factor, plays an important role in determining human behaviour, particularly when being physically active [47]. Both types of motivation, i.e., internal [intrinsic] such as belief within oneself and external [extrinsic] such as social rewards, influence PA levels [66]. This current research study found that motivation was a central theme throughout the reflections, with participants discussing how difficult it was to maintain motivation over time. Participants (*n* = 7 out of 11, 64%) expressed varying types of motivation toward PA. Those with intrinsic motivation described a personal desire to stay active and achieve fitness goals for reasons such as enjoyment, self-discipline and a sense of accomplishment. Others (*n* = 6 out of 11, 55%) demonstrated extrinsic motivation, relying on factors such as praise from peers, feedback from coaches or structured support systems to remain committed. The role of self-motivation was also highlighted, with participants indicating that once they acknowledged the positive impacts of exercise on their lives, they could motivate themselves to return to regular PA. These results are similar to previous studies where it was reported that demotivation among young adults stemmed from apprehension about PA, laziness, lack of interest, negative emotions such as resentment and a dislike of being observed while being physically active or exercising alone [67]. In addition to this, the lack of social support from friends and family was reported as a prevalent factor contributing to demotivation [62]. However, students from this study mentioned that online resources, such as fitness videos or articles, were frequently cited as convenient sources of information that helped participants stay engaged with their fitness goals. A plausible reason for this may be because social networking sites [such as YouTube, TikTok and Instagram] are popular among young adults such as undergraduate university students [68]. Social media is a beneficial tool for mass communication and thus, students are motivated to be physically active when viewing exercise content online [69]. Therefore, it is important to place the focus on motivation as a psychosocial factor that could either enhance or hinder PA participation and consequently develop interventions that are relevant and suitable to the contemporary student [18,70].

### 4.5. Strengths and Limitations

This study aimed to explore students’ PA participation, by providing insights into the psychosocial factors that shape their experiences. A core strength of the use of I-poems, as a methodological approach within qualitative research, is the ability to highlight the multi-dimensionality of the subjective experiences of undergraduate university students. Each participant’s experience was brought to life through the use of an I-poem. The poems depict positive, negative and neutral feelings of the subjective experiences of the students regarding PA engagement and psychosocial factors. This ultimately provides a deeper understanding of ‘what is actually going through a student’s mind and how they are feeling’. This approach is beneficial, as other types of research methodologies, such as quantitative studies, would not have been able to replicate it. However, this research study is not without faults and thus, limitations need to be acknowledged. Firstly, this study included a convenient sample of 18 undergraduate students from a single South African university. While the use of I-poems provided deep insights into students’ psychosocial journeys and PA behaviours, the sample size and institutional context may restrict the generalisability of the findings. Thus, the experiences captured may not fully reflect those of students in other universities or socio-cultural environments. A limitation of this study is the predominance of female participants, as well as a substantial representation of students from sport-related departments. Future research should aim to include students from different departments to capture a broader and more diverse range of perspectives on PA. Additionally, since I-poems focus on the personal experiences of individual participants, the findings from this study may not be generalisable to larger populations. The insights gained are specific to the participants involved, limiting the broader applicability of the results. To address this limitation, future researchers could expand the sample size and include participants from different demographic backgrounds or institutions, increasing the potential for more widely applicable insights while maintaining the depth of personal experience provided by I-poems. Secondly, the analysis of I-poems in this study was time-consuming and complex, requiring a detailed, in-depth examination of individual expressions. This process involved identifying patterns across multiple participants’ subjective experiences, which could be resource-intensive and challenging to manage effectively. Future researchers could consider employing a team of analysts to help manage the volume of data and provide diverse perspectives in the interpretation of the I-poems, potentially making the analysis process more efficient and comprehensive. Future studies wishing to conduct similar research should consider exploring I-poems in conjunction with other research methodologies to provide a more robust explanation of students’ PA participation and psychosocial factors [30].

### 4.6. Practical Applications and Recommendations

The findings of this study offer valuable implications for university policies and student support services aiming to enhance participation in PA. Institutions should consider implementing student-centred and emotionally responsive interventions that integrate both physical and mental health components. For example, structured programmes such as “Mindful Movement” sessions—which combine light PA with reflective practices like guided journaling or group discussions—can address students’ psychosocial needs alongside promoting PA. In addition, peer-led initiatives, where trained student ambassadors co-design and facilitate activities that resonate with their peers’ lived experiences, may foster greater engagement. Embedding psychosocial support within PA initiatives, such as incorporating mindfulness techniques, breathing exercises or informal mental health check-ins, can further support students who experience stress, anxiety or emotional fatigue. Finally, campus-wide campaigns that feature student narratives, including excerpts from I-poems, can help cultivate a supportive culture around mental wellness and physical activity. These recommendations provide a foundation for universities to align health promotion strategies with the lived realities and expressed needs of their undergraduate student population.

## 5. Conclusions

This study aimed to explore students’ PA participation, by providing insights into the psychosocial factors that shaped their experiences. By employing the Listening Guide and generating I-poems from the qualitative data, undergraduate university students’ subjective experiences and perspectives were explored. This study underscores the utility of I-poems as an innovative qualitative approach for exploring the psychosocial factors of PA among undergraduate students. By focusing on the participants’ voices, the findings offer insights into the complex interplay of mental health, motivation and social support in shaping the PA journey of a student. The results highlight the importance of incorporating psychosocial considerations into the design of contextually relevant, student-focused interventions to foster sustained PA participation. These findings contribute to advancing qualitative inquiry in PA research and provide valuable perspectives for health promotion strategies targeting young adults. This ultimately contributes to advancing Sustainable Development Goal 3, which focuses on good health and well-being.

## Figures and Tables

**Table 1:** Interview schedule.

*Category*	*Interview Questions and Probe Questions*
*PA*	What do you understand by the term PA? What is the daily/everyday lifestyle of a university student? Is being physically active a positive or negative aspect? Why? What influence do you think PA has on your life? (What type of influence is that, do you think, for example, good or bad?) Are you physically active? If not, why not? If yes, what helps you to be physically active? If you are physically active, what type of activities would you participate in? Why? If you are not physically active, what would you like to participate in? What are the barriers to being physically active? (What restricts you from being physically active?) What are the facilitators of being physically active? (What enables you to be physically active?)

Note: PA = physical activity.

**Table 2:** Demographic information of participants.

POEM NUMBER	PSEUDONYM	GENDER	AGE	YEAR OF STUDY	DEPARTMENT STUDENT IS STUDYING IN
**1**	Cassidy	Female	21	3	Sport, recreation and exercise science
**2**	Nozi	Female	21	2	Physiotherapy
**3**	Chelsea	Female	20	2	English and sociology
**4**	Zanele	Female	23	3	Social work
**5**	Gabby	Female	20	2	Sport, recreation and exercise science
**6**	Rose	Female	19	2	Biotechnology
**7**	John	Male	21	3	Physiotherapy
**8**	Zinzi	Female	19	3	Nursing
**9**	Matthew	Male	20	1	Social work
**10**	Aphiwe	Female	21	4	Industrial psychology
**11**	Thandi	Female	22	4	Industrial psychology

**Table 3:** Themes generated from the I-poems.

Main Theme	Subtheme(s)
PA	Types of activities Consistency and barriers to participation The role of enjoyable and accessible activities Influence of physical activity on overall fitness and routine
Mental health	Mental health challenges (depression, anxiety, stress) PA as a coping mechanism for mental health Influence of body image and self-esteem on motivation to be active Emotional regulation and mood improvement through PA
Motivation	Setting goals and accountability structures Influence of external motivators Barriers influencing motivation Use of digital resources and self-reliance in maintaining motivation
Social Support	Importance of friends, family and peers for encouragement and accountability Role of social groups and clubs in fostering PA participation Feelings of isolation and trust issues hindering social support Influence of social media and peer networks on motivation and connection

Note: PA = physical activity.

## Data Availability

The data and supportive information are available within the article.

## Abbreviations

The following abbreviations are used in this manuscript:

ADHD	Attention-deficit/hyperactivity disorder
COVID-19	Coronavirus-19
PA	Physical Activity
SA	South Africa
WHO	World Health Organisation
